# Advancements in nanoparticle-based vaccine development against Japanese encephalitis virus: a systematic review

**DOI:** 10.3389/fimmu.2024.1505612

**Published:** 2024-12-20

**Authors:** Takele Adugna, Qingli Niu, Guiquan Guan, Junzheng Du, Jifei Yang, Zhancheng Tian, Hong Yin

**Affiliations:** ^1^ State Key Laboratory for Animal Disease Control and Prevention, Lanzhou Veterinary Research Institute, Chinese Academy of Agricultural Sciences, Lanzhou, China; ^2^ Jiangsu Co-innovation Center for Prevention and Control of Important Animal Infectious Diseases and Zoonoses, State Key Laboratory of Veterinary Etiological Biology Project, Yangzhou, China; ^3^ College of Veterinary Medicine and Animal Sciences, University of Gondar, Gondar, Ethiopia

**Keywords:** Japanese encephalitis, Japanese encephalitis virus, nanoparticle, systematic review, vaccine

## Abstract

Vaccination remains the sole effective strategy for combating Japanese encephalitis (JE). Both inactivated and live attenuated vaccines exhibit robust immunogenicity. However, the production of these conventional vaccine modalities necessitates extensive cultivation of the pathogen, incurring substantial costs and presenting significant biosafety risks. Moreover, the administration of live pathogens poses potential hazards for individuals or animals with compromised immune systems or other health vulnerabilities. Subsequently, ongoing research endeavors are focused on the development of next-generation JE vaccines utilizing nanoparticle (NP) platforms. This systematic review seeks to aggregate the research findings pertaining to NP-based vaccine development against JE. A thorough literature search was conducted across established English-language databases for research articles on JE NP vaccine development published between 2000 and 2023. A total of twenty-eight published studies were selected for detailed analysis in this review. Of these, 16 studies (57.14%) concentrated on virus-like particles (VLPs) employing various structural proteins. Other approaches, including sub-viral particles (SVPs), biopolymers, and both synthetic and inorganic NP platforms, were utilized to a lesser extent. The results of these investigations indicated that, despite variations in the usage of adjuvants, dosages, NP types, antigenic proteins, and animal models employed across different studies, the candidate NP vaccines developed were capable of eliciting enhanced humoral and cellular adaptive immune responses, providing effective protection (70–100%) for immunized mice against lethal challenges posed by virulent Japanese encephalitis virus (JEV). In conclusion, prospective next-generation JE vaccines for humans and animals may emerge from these candidate formulations following further evaluation in subsequent vaccine development phases.

## 1 Introduction

**Figure 1:**
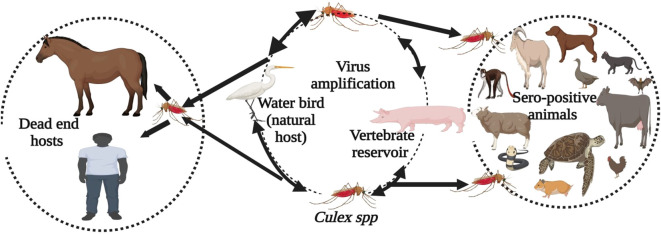
Transmission cycle of the Japanese Encephalitis Virus. Culex mosquitoes, along with amplifying hosts such as pigs and wading birds like waterfowl, maintain the enzootic transmission cycle. Humans and horses are considered dead-end hosts. In domestic and wild mammals, poultry, reptiles, and amphibians, JEV infection has been identified based on direct evidence (virus, viral antigen, or viral RNA) and indirect evidence (antibody) (5).

The Japanese encephalitis virus (JEV) is a notable member of the mosquito-borne viruses in the Flaviviridae family, responsible for the most prevalent form of encephalitis in humans and horses across the Asia-Pacific region (1). This region includes approximately 24 countries, including the two most populous nations, China and India, as well as several densely populated countries such as Singapore and Bangladesh (2, 3). JEV is primarily transmitted by mosquitoes of the genus *Culex* (4). Pigs and wading birds play key roles in maintaining viral circulation and transmitting the virus to humans and horses (**Figure 1**) (6, 7). Annually, an estimated 68,000 to 100,000 clinical cases of Japanese encephalitis (JE) occur worldwide, resulting in approximately 15,000 to 25,000 fatalities (8, 9). JEV is emerging in new areas, with reported cases in Europe and Africa (10, 11).

Distinguishing JE from other encephalitis cases is challenging, making laboratory confirmation essential (12). The WHO recommends diagnosing JE using the IgM-capture ELISA (MAC-ELISA) from a single cerebrospinal fluid (CSF) or serum sample, with CSF preferred to reduce false positives from prior infections or vaccinations (13). Other tests, such as the hemagglutination inhibition (HI) test, indirect immunofluorescence assay (IFA), plaque reduction neutralization test (PRNT), and nucleic acid detection, may also be used (14, 15). There is no specific antiviral treatment for JEV; management is supportive, focusing on nutrition, airway maintenance, and anticonvulsants for seizure control (16–18). Vaccination can effectively prevent JEV infection in both humans and animals (19, 20).

Live attenuated and inactivated vaccines against JE have been available for decades (**Table 1**), yet JEV remains the leading cause of viral encephalitis in Asia (23). Approximately 3 billion people in endemic regions are at risk, but only 80–100 million JE vaccine doses are produced annually, highlighting a significant gap in coverage (21). Vaccination costs range from $1.15 to $2.41 per child per dose (24–26). A study in the Philippines found that managing JE illness can cost between $859 and $1,209 per case. JE’s impact drives vaccine demand, increases healthcare costs, and emphasizes the need for public health initiatives and further vaccine development. Its spread into new territories also raises global health security concerns, prompting greater international collaboration and funding for surveillance and response (24, 27).

**Table 1:** Licensed Japanese encephalitis vaccines (21, 22).

Vaccine type	Substrate	Trade name	Vaccine strain	Licensing year and country
Inactivated	Mouse brain	BIKEN^®^, JE-VAX, Sanofi Pasteur	Nakayama strain Beijing-1 strain	Japan in 1954
	Hamster kidney cells		Beijing-3 or P-3	China in 1968
	Vero cells	JEBIK^®^	Beijing-1	Japan in 2009
		ENCEVAC^®^	Beijing-1	Japan in 2011
		JEVACTM	Beijing P-3 strain	China in 2008
		IXIARO^®^ (USA, EU); JESPECT^®^ (AUS, NZ); JEEV^®^	SA-14-14-2	USA, Australia, and Europe in 2009
		JENVAC^®^	Kolar821564XY	India in 2014
Live attenuated	Hamster kidney cells	CD.JEVAX^®^	SA-14-14-2	China in 1988
Chimera	Vero cells	IMOJEV^®^	JE SA-14-14- 2/Yellow fever 17 D	Australia and Thailand in 2012

Live-attenuated vaccines induce strong immune responses but can be risky for immunocompromised individuals (28), while whole inactivated vaccines are safer but often have short-lived efficacy (29). Both require large-scale pathogen cultivation, leading to high costs and biosafety concerns (28, 30). Furthermore, traditional JEV vaccines offer limited cross-protection against different strains due to the virus’s genetic variability, undermining their efficacy against emerging variants (15, 30). Consequently, there is an urgent need to develop a safe, stable, durable, and cost-effective vaccine that elicits robust immunogenicity across diverse JEV strains. Recombinant pathogen-derived vaccines, coupled with nanoparticle (NP) delivery systems, present a promising solution to meet this critical need (31).

Nanoparticle vaccines represent a breakthrough in vaccinology, offering enhanced immune responses, safety, stability, and adaptability. While research is ongoing, these vaccines show significant promise in addressing a wide range of infectious diseases more efficiently than traditional vaccines. NPs inherently contain pathogen-associated molecular patterns (PAMPs) that are recognized by the immune system as signals of potential danger, thereby improving antigen presentation and enhancing immune responses (32). Additionally, nanoparticles can carry multiple antigens, enabling the development of multivalent vaccines. Types of NPs, such as protein nanocages, outer membrane vesicles (OMVs), virus-like particles (VLPs), and polymeric NPs, effectively overcome barriers to recombinant vaccine delivery (31).

During NP vaccination, innate immune cells, such as antigen-presenting cells (APCs) like dendritic cells (DCs) and macrophages, detect and engulf NPs, triggering antigen presentation on MHC Class I and II molecules and activating T-helper (CD4+) and cytotoxic (CD8+) T cells (31–35). CD4+ T cells promote B-cell differentiation and antibody production, which inhibit viral replication (36), while CD8+ T cells eliminate infected cells when humoral immunity is insufficient (36–38). After endosomal uptake, NPs release antigens into the cytoplasm, activating CD8+ T cells to target and destroy infected cells (39). NP delivery systems also stimulate T- and B-cell memory, contributing to a long-lasting immune response (40). Recombinant vaccines are particularly advantageous for incorporating a limited set of B- and T-cell epitopes, as their ability to precisely engineer specific immunogenic components results in targeted, efficient immune activation (41). By focusing on well-characterized epitopes conserved across JEV strains, developers can elicit stronger immune responses, reduce immune tolerance, and potentially achieve cross-protection against diverse strains (31). Encapsulating these epitopes in NPs further enhances immune responses, minimizes adverse effects, and enables cost-effective production (42).

Nanoparticle vaccine technology has progressed from laboratory research to clinical application. Outer membrane vesicles (OMVs) and virus-like particles (VLPs) have received approval from the Food and Drug Administration (FDA) and are currently available on the market, while additional vaccines are under investigation for safety and efficacy (43). Researchers are developing a nanoparticle vaccine for JEV, with promising results, though it has not yet reached clinical trials or FDA approval. This review consolidates findings on nanoparticle vaccine development for JEV, with the aim of enhancing understanding and guiding future research.

## 2 Methods

### 2.1 Literature search and study selection

**Figure 2:**
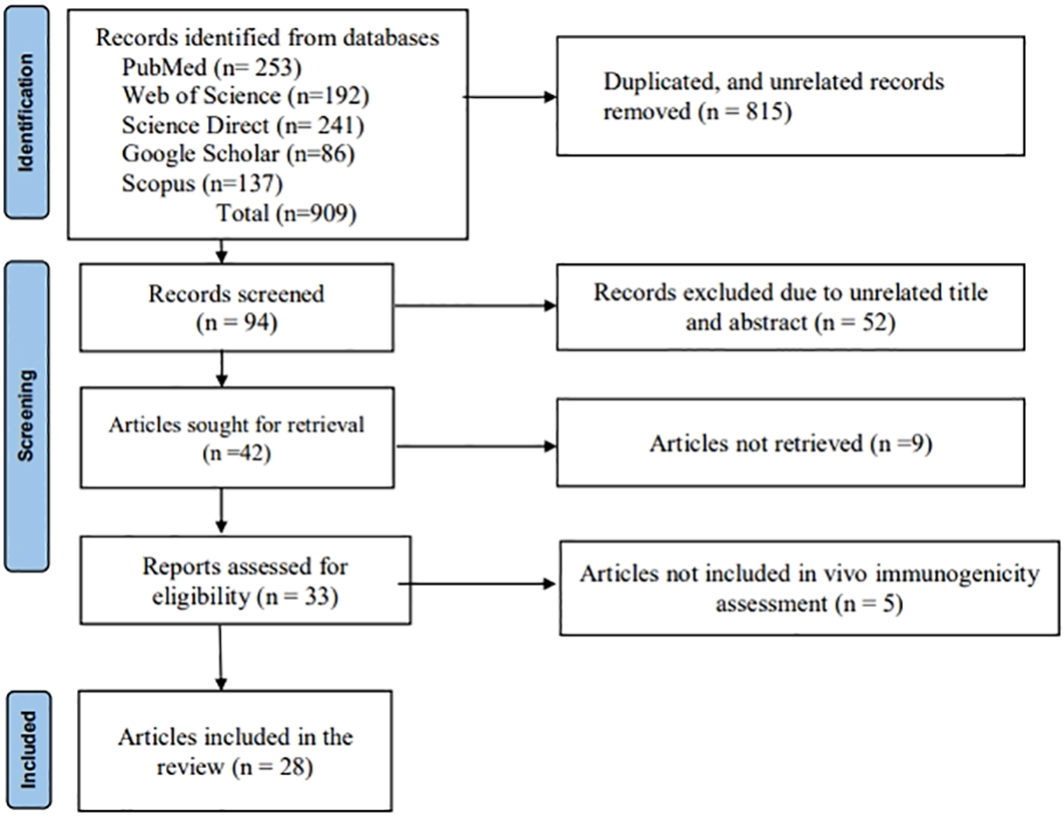
Article searching and screening flow diagram of the systematic review (PRISMA 2020).

The methodology outlined in the PRISMA (Preferred Reporting Items for Systematic Reviews and Meta-Analyses) declaration was followed in the design and preparation of this systematic review (44). English-language databases, including PubMed, Web of Science, ScienceDirect, Google Scholar, Scopus, and EBSCO (**Figure 2**), were systematically explored for research articles published between 2000 and 2023 that focused on the development of candidate nanoparticle vaccines against JEV with *in vivo*/animal model immunogenicity evaluation. Keywords such as “Japanese encephalitis,” “nanoparticle,” “virus-like particle,” “self-assembled particle,” “immunogenicity,” “antigenicity,” “vaccine,” and “candidate vaccine” were included in the comprehensive search. Additional searches were conducted by reviewing the bibliographies of relevant primary and review publications. Furthermore, a thorough manual search of the papers’ full reference lists was carried out to ensure no articles were overlooked. Full-text articles published in the aforementioned databases and those evaluating the proposed vaccine *in vivo*/animal models were included. We excluded gray literature and abstracts of papers presented at conferences. After a thorough evaluation of the articles, studies were excluded if they met any of the following criteria: (1) studies that did not focus on vaccine candidates that form nanoparticles; (2) studies that were not available in full text; (3) studies not published in English; (4) reviews or descriptive studies; and (5) articles that lacked sufficient information about the vaccine’s immunogenicity and level of protection in an animal model.

### 2.2 Data extraction

The types of nanoparticles (NPs), antigenic components, expression systems, virus strains used for challenges, NP sizes, doses for single immunization, adjuvants, number of booster doses, animal models, routes of administration, positive control vaccines, methods for assessing immunogenicity, and key results regarding immunogenicity were systematically compiled in a well-structured data extraction Excel sheet. Additionally, bibliographic details, including authors and publication years, were documented.

## 3 Results

### 3.1 Analysis of the included literature

**Figure 3:**
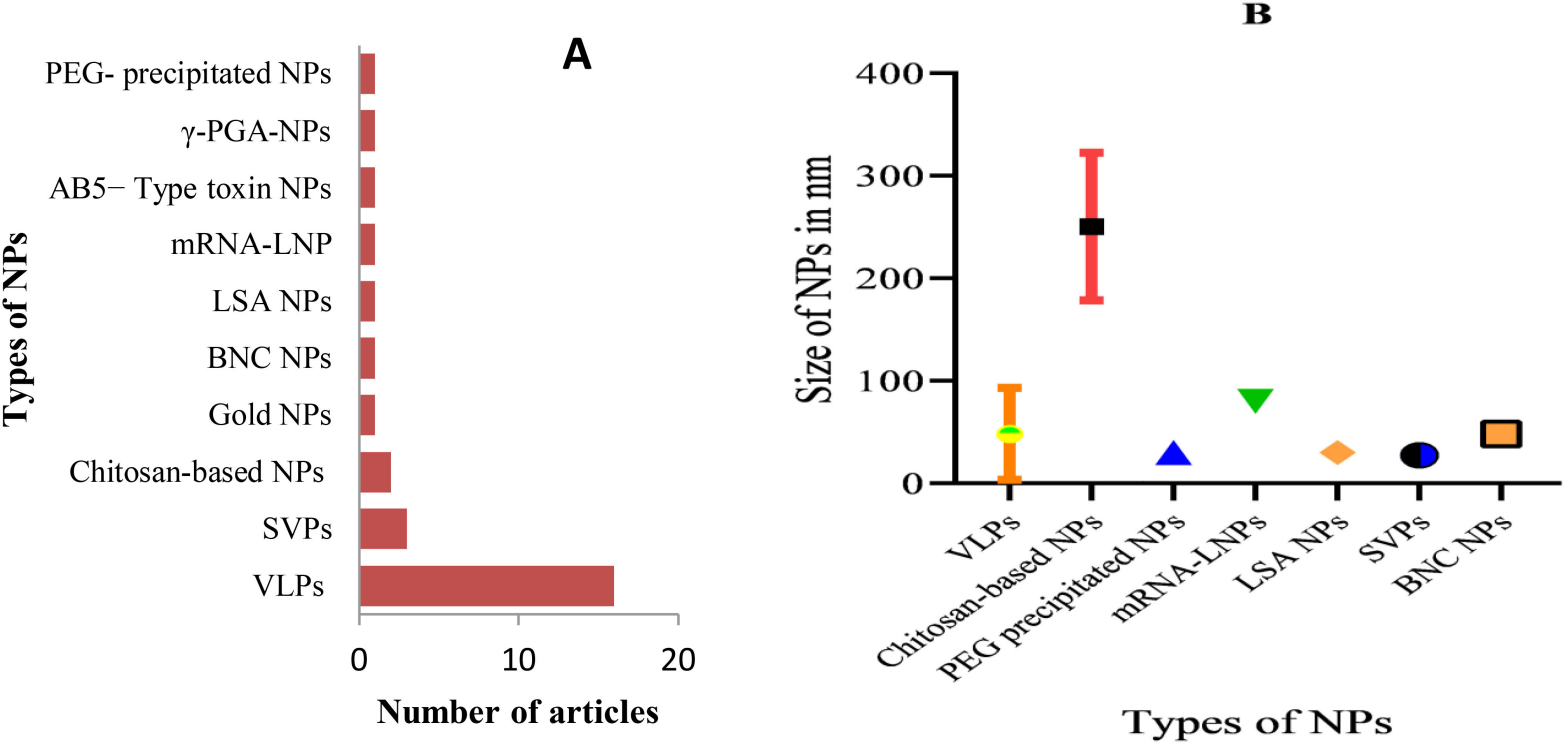
Types of developed JEV NP vaccines and measured particle size: **(A)** Types of developed anti-JEV NP vaccines and the frequency of studies; **(B)** Size range of developed NPs in nanometers, categorized by their respective NP type. PEG, Polyethylene glycol; LSA, Lumazine synthase-assembled; BNC, Bio-nanocapsules.

This systematic review included a total of 28 articles (**Figure 2**) published from 2001 to 2023 that studied potential candidate vaccine development against JEV by incorporating particulate antigens into NPs. Different NP formation techniques were employed in these studies (**Table 2**, **Figure 3A**): 16 VLPs, 3 Sub viral particles (SVPs), 2 chitosan-based NPs, and other NPs, including one study each on colloidal gold, bio-nanocapsules, lumazine synthase-assembled particles, lipid-based nanoparticles (LNPs), AB5-type toxin-based nanoscaffolds, γ-PGA, and polyethylene glycol-precipitated NPs.

**Table 2:** Nanoparticle-based JE vaccine development studies and their results.

Nano vaccine complex	Antigen	Expression host	Delivery system	Animal model	Positive control	Immunogenicity assessment	Main result	Reference
VLPs	prM and E proteins	BHK	S.C., 4μg with Montanide ISA50V2 adjuvant	BALB/c	Attenuated JEV (SA14-14-2)	NAbs assay Lethal dose challenge	Strong NAbs 100% protection	(45)
VLPs	E protein	Silkworm pupae	Unspecified dose and route	BALB/c	------	NAbs assay	High NAbs titer against the Muar and Nakayama strains Lower NAbs titer against the Beijing-1	(46)
				Rabbit				
Colloidal gold	prM and E proteins DNA	---------	I.V., & I.D. 0.5μg	BALB/c	Inactivated JEV vaccine	NAbs assay Lethal dose challenge	Speeds up the production of NAbs I.V. and I.M. inoculation led to more rapid and pronounced NAbs production. Both active and passive immunization confer 100% protection	(47)
VLPs	E protein	*E. coli*	S.C., 10μg without adjuvant	FVB/J	------	NAbs assay Lethal dose challenge	Induced higher NAbs. 100% protection against intracerebrally inoculated lethal dose challenges.	(48)
VLPs	prM and E proteins	RK13	I.P., 503 ELISA equivalent dose without adjuvant	BALB/c	Inactivated JE-VAX	NAbs assay Lethal dose challenge p	Exhibited high levels of NAbs, as did the licensed JE-VAX. Complete protection	(49)
VLPs	prM and E proteins	HS-deficient CHO cel**l**	S.C., 0.04μg with Freund’s adjuvant	BALB/c	------	Immunoglobulin (Ig) assay NAbs assay Lethal dose challenge	Three doses produced effective NAbs and neutralized homologous GI and heterologous GIII High level of IgG titers, 100% and 90% protection against GI and GIII challenges.	(50)
			S.C., 5μg with Freund’s adjuvant	Swine				
Chitosan -based NPs	live attenuated JE chimeric virus vaccine	-------	I.N. & S.C., 104 PFU using Chitosan (CS) or Chitosan maleimide (CM)	C57BL/6	Live attenuated JE-CV	NAbs assay Ig antibody assay Cytokine assay	S.C. injection elicited significantly higher NAbs and cytokine (IL-4 and IFN-ɣ) levels. I.N. immunization only induced a higher specific anti-JEV IgA level.	(51)
VLPs	E protein	*E. coli*	I.M., 20 μg with Adju-phos adjuvant	BALB/c	JEV vaccine	NAbs assay	Induced considerably higher NAbs	(52)
SVP	prM and E proteins	RK13	S.C., 1μg with Freund’s adjuvant	ICR	Inactivated Nakayama	NAbs assay	Induced NAbs following two immunizations	(53)
SVP	prME and E2 of HVC	HEK-293T	I.P., 20μg with Alum or CpG	BALB/c	--------	Ig assay NAbs assay	Induced anti-JEV IgG It abled to neutralize *in-vitro* infection	(54)
γ-PGA based NPs	Inactivated JEV	------	I.P., 1μg with γ-PGA as an adjuvant and NPs forming unite	BALB/c	Inactivated vaccine (JE BIKEN)	NAbs assay Lethal dose challenge	Using γ-PGA-NPs as an adjuvant boosted 10 times higher NAb titers than the inactivated vaccine alone. A single dose with and without γ-PGA-NPs had 100% and 50% protection, respectively. Aluminum adjuvant also showed similar levels of effectiveness.	(55)
VLPs	E protein	RK13	I.P., 0.3μg without adjuvant	ddY	Inactivated virions (JE-VAX)	NAbs assay Lethal dose challenge	Induced NAb titers, as high as the licensed JE vaccine. Total protection against challenges	(55)
PEG precipitated Particle	prM and E proteins	COS-1 cell	S.C., 1μg (virus equivalent) with Freund’s adjuvant	ICR	JE-VAX	NAbs assay	Elicited the highest NAbs titers	(57)
VLPs	CprME proteins	HEK-293T	I.M., 1μg with Alum adjuvant	BALB/c		NAbs assay	High levels of neutralizing antibodies Abled to induce strong NAb titers when combined as a tetravalent vaccine against four viruses (Zika virus (ZiKV), Chikungunya (cHiKV), Yellow fever (YfV), and JEV)	(58)
VLPs	prM and E proteins	Lepidopteran insect cells	I.M., 10μg with Alum adjuvant	C3H/HeN		NAbs assay	Induced NAbs	(59)
AB5-Type toxin based nanoscaffold	ED-III Protein	*E. coli*	I.V., 25μg with Alum adjuvant	Balb/c	Inactivated JEV VAX	Ig assay NAbs assay	Induced high total IgG level and similar IgG1 levels with inactivated vaccine. Induced comparable Nab titers with that of inactivated vaccine.	(60)
VLPs	prM and E proteins	AP-61 cells	S.C., 1μg without adjuvant	BALB/c	IMOJEV (live, attenuated)	Ig assay NAbs assay Cytokine assays	Produced comparable Nab titers against GI and GIII and substantially greater levels of specific IgG, IgG1, and IgG2a antibodies than licensed vaccines. Enhanced IFN- and IL-4 production	(61)
mRNA-LNP	prM and E proteins	HEK-293T	I.M., 15μg without adjuvant	C57BL/6	SA14-14-2 (live-attenuated vaccine)	NAbs assay Lymphocyte proliferation assay Lethal dose challenge	Induced efficient Nabs. Elicited strong CD8+ T cells Secreted higher IFN-γ level Protected 100% of the challenged mice	(62)
Lumazine synthase assembled NPs	ED-III Protein	*E. coli*	S.C., 0.04μg with Montanide IMS-1313 adjuvant	BALB/c	SA14-14-2	Ig assay NAbs assay Cytokine assays Lymphocyte proliferation assay Lethal dose challenge	Higher level of cytokines (IFN-α, IL-12, and IFN-γ), and IgG1 and IgG2a antibodies were secreted. Induced strong NAbs The IgG1 and IgG2a levels hadn’t significant difference between the EDIII-LS and SA14-14-2 groups. 100% protection was offered by EDIII-LS and SA14-14-2 vaccines. Whereas the ED III subunit vaccine had a 55% survival.	(63)
VLPs	E protein	RK13	I.P., 1μg with γ-PGA-NPs or Alum	BALB/c	Inactivated JE-VAC	NAbs assay Lethal dose challenge	Using γ -PGA NPs as adjuvants induced significantly higher titers of NAb NAb level of JE-VAC plus γ -PGA NPs were significantly higher than that of JE-VLP plus γ -PGA NPs. Antibody titers in the serum were maintained for 6 months. Single-dose immunization of JE-VLP and JE VAC with either γ -PGA-NPs or alum protected 90% and 100% of the challenged mice, respectively. Single-dose JE-VLP or JE VAC without adjuvant confers only 20% protection.	(64)
SVP	E protein	C6/36 cells & BHK-21	I.P., 0.4μg without adjuvant	ICR	Inactivated JE VAX	Ig assay NAbs assay Lymphocyte proliferation assay	Produced detectable level of antibody against Nakayama strain and any of the examined JEV GV strains, except R84K, whereas JEVAX didn’t develop for any of the GV JEV strains. A mixture of JEVAX with Mu-SVP led to the production of higher NAbs against GV and GIII. Th2-dominant cellular responses observed	(65)
VLPs	prM/E protein	Silkworm Bm-N cells	Unspecified route, 20μg with Freund’s adjuvant	Unspecified mice		NAbs assay	NAbs against the Nakayama, Beijing-1, and Muar strains were induced. A higher tendency to react with the Muar strain than the Nakayama strain was observed.	(66)
			Unspecified route, 40μg with Freund’s adjuvant	Rabbit				
VLPs	prM and E proteins	HEK-293T	I.P., 5log10 without adjuvant	BALB/c and C57BL/6	-------	NAbs assay	Elicited NAbs against GI, GII, and GV strains Induced higher IgGs A second dose after a month of the prime injection greatly boosted antibody titers Immunization of piglets with two doses induced high titers of antibodies	(67)
			I.M., 6log10 without adjuvant	Swine				
VLPs	prM/E	Yeast cell (Pichia pastoris strain X33)	S.C., 10μg without adjuvant	BALB/c	SA 14-14-2	NAbs assay Lymphocyte proliferation assay Lethal dose challenge	Induced a similar NAbs level to that of the live vaccine. Induced IgG antibodies more rapidly than the live vaccine. T-cell response was lower than that of SA 14-14-2 Passive transfer of VLP antisera protects 100% of challenged Induces a protective immune response in pigs.	(68)
			I.M., 20μg without adjuvant	Swine				
Chitosan-based NPs	DNA of prM and E protein gene	—–	I.D,. 10μg with Cytosine phosphoguanine (CpG) adjuvant	C3H/HeN	-------	NAbs assay Lethal dose challenge	Elicit desired specific NAbs Conferred 100% and 50% protection With and without adjuvants, respectively.	(69)
VLPs	E protein	*E. coli*	S.C., 50μg with Freund’s adjuvant	BALB/c	SA14-14-2	NAbs assay Cytokine assay Lymphocyte proliferation assay Lethal dose challenge	Induced NAbs and cytokine level however it was significantly lower than from SA14-14-2 vaccine Both the candidate and positive control vaccines protected 100% of the challenged mice	(70)
VLPs	ED-III Protein	*E. coli*	S.C., 34μg with Alum adjuvant	BALB/c	IMOJEV	Ig assay Cytokine assay Lymphocyte proliferation assay	Exhibited a higher specific anti ED-III antibody titer than IMOJEV Triggered the proliferation of cytotoxic T-lymphocytes, macrophages, and natural killer (NK) cells. Induced high proliferation of CD3^+^CD4^+^ and CD3^+^CD8^+^ T-cells	(71)
Bio-nanocapsule based NPs	ED-III Protein	*E. coli*	S.C., 30μg with Alum adjuvant	BALB/c	inactivated vaccine	NAbs assay Lethal dose challenge	Induced NAbs With and without adjuvant it provided 70% and 44.4% protection, respectively	(72)

### 3.2 Size and antigenic components of NPs

The primary goal of developing nanoparticle-based vaccines is to deliver antigenic components that structurally and functionally resemble live pathogens. These antigens cannot replicate or cause infection, but they contain immunogenic elements of the pathogen that can be recognized by APCs and are essential for fully activating the immune system (43). Therefore, it is commonly understood that particles containing antigenic components and sized similarly to viruses are recognized by the body as viruses. Seven of the 28 compiled articles did not specify the size of the developed NPs. The size of the NPs varied significantly across different antigen-particulate approaches used in the articles. For example, chitosan-based NPs ranged from 200–333 nm, while VLPs and sub-viral particles ranged from 18–200 nm and 25–30 nm, respectively. The remaining NP types, which ranged in size from 30 to 80 nm, were used in only one study. To better understand the variations in NP size, the error bar (**Figure 3B**) provides an overview of how nanoparticle sizes differ across various antigen-particulate techniques.

**Figure 4:**
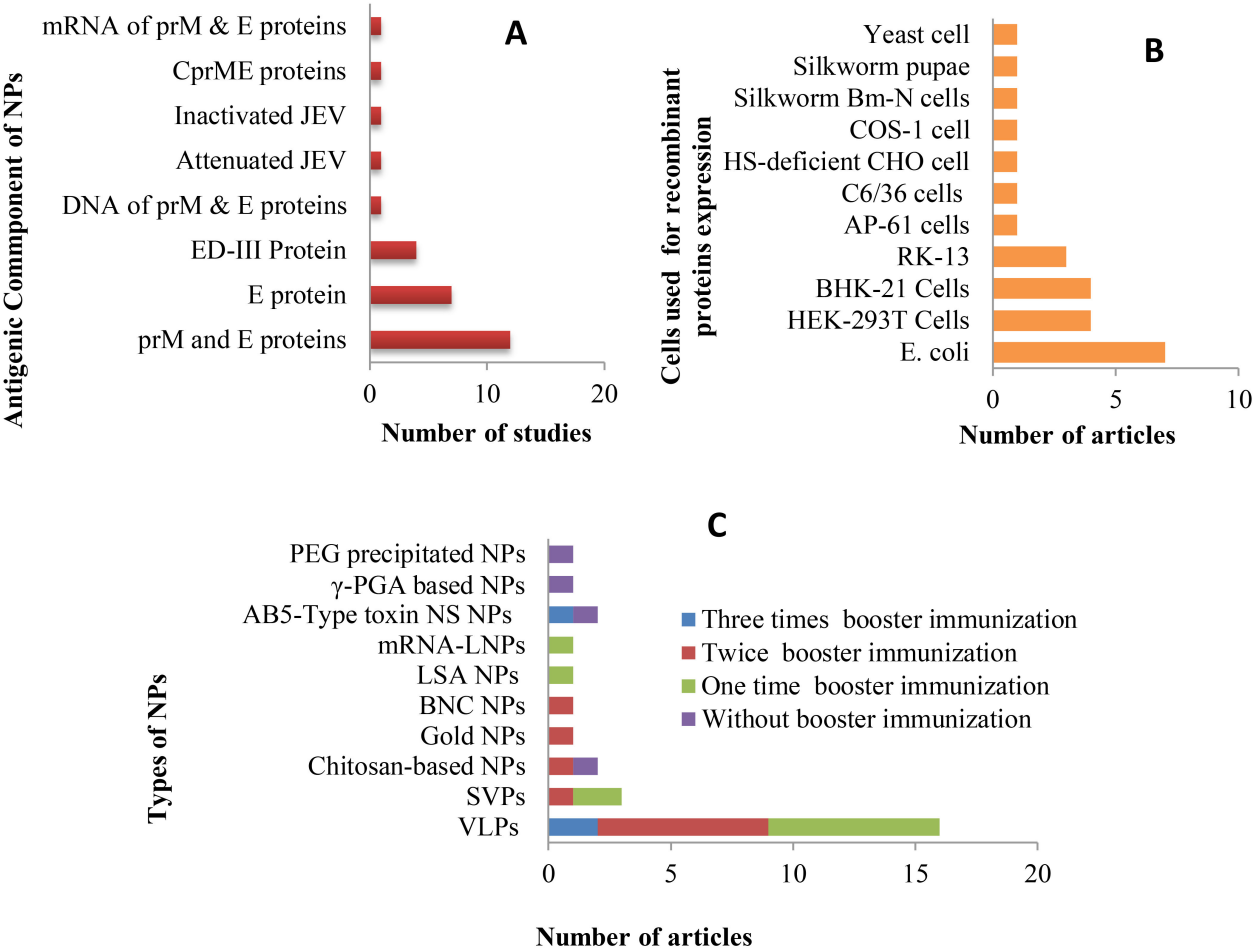
Antigenic components, expression hosts, and number of booster administrations applied in studies on new JE NP vaccine development: **(A)** Antigens and their corresponding study frequencies; **(B)** Expression hosts used for the production of target antigenic proteins and their corresponding study frequencies; **(C)** Types of developed NP vaccines and the number of booster immunizations administered, along with the frequency of studies.

Nanoparticles serve as carriers to facilitate the attachment of antigens to their surface, thereby enhancing immune response by promoting efficient trafficking and recognition by cellular receptors (73). Structural proteins, mRNA or DNA encoding these proteins, as well as inactivated or live whole pathogens, were utilized as antigenic components in the development of NP-based vaccines in the studies reviewed (**Figure 4A**). Among the 28 publications examined, Pre-membrane and Envelope (prM and E) combined protein, E protein, and E domain-III protein were employed in 12 (42.86%), 7 (25%), and 4 (14.3%) studies, respectively. Additionally, single articles featured the use of Capsid, Pre-membrane and Envelope (CprME) proteins, mRNA of prM & E proteins, DNA of prM & E proteins, attenuated JEV, and inactivated JEV as antigenic components.

### 3.3 Expression systems of the recombinant protein

The expression of recombinant proteins is a pivotal element in the development of antigenic protein-based NP vaccines. Over the years, recombinant protein expression has been successfully carried out in a variety of host systems, including yeasts, bacteria, plants, transgenic animals, as well as cultured insect and mammalian cells (74). In the studies reviewed, *E. coli*, HEK-293T, BHK-21, and RK-13 cells were employed to express recombinant JEV antigenic proteins, with respective study frequencies of 7, 4, 4, and 3. Additional expression systems used in individual studies included yeast (*Pichia pastoris* strain X33), silkworm Bm-N cells, COS-1, HS-deficient CHO cells, C6/36, and AP-61 cells, as well as silkworm pupae (**Figure 4B**).

### 3.4 Laboratory animals

Mice are the most commonly used animals in JEV vaccine development and pathogenesis studies, despite variations in their susceptibility depending on factors such as viral strain, inoculum dose, route of administration, and age (75, 76). All studies included in this review used mice as the animal model; however, swine and rabbits were also utilized alongside mice in 3 and 2 studies, respectively. A total of six specified mouse strains, as well as one unspecified strain, were employed across the studies (see **Table 2**). BALB/c mice were used in the majority of studies (19), while C57BL/6 and ICR strains were each used in 3 studies, and C3H/He, ddY, and FVB/J strains were used in 2, 1, and 1 study, respectively.

### 3.5 Dosage and routes of administration

For new vaccine candidates, dose-response studies are often conducted to determine the optimal dose for inducing the highest antibody production in animal models. Several studies (45, 47, 50, 61, 67, 68) included in this review carried out dose-optimization experiments at the animal model level during the development of NP-based JEV vaccines. Another key aspect of dosage evaluation is determining whether a single immunization or additional booster doses, along with adjuvants, are necessary to elicit a protective immune response. In nanoparticle-based vaccines, synthetic nanoparticles, such as inorganic and liposome-based particles, typically require multiple doses and the addition of adjuvants, while biologically derived nanoparticles often have intrinsic properties that enhance their ability to stimulate the immune system independently, reducing the need for artificial adjuvants (43). Consequently, many of the studies reviewed focused on self-assembled VLPs and sub-viral particles, which required booster immunizations and the use of adjuvants. Of the 28 articles reviewed, 4 studies used only a single-dose immunization, while 7 studies did not incorporate adjuvants. The remaining studies administered 1–3 booster doses (**Figure 4C**) and utilized various adjuvants, including Alum, Freund’s, Montanide ISA50V2 & IMS-1313, and Cytosine-phosphoguanine (CpG).

The adjuvanticity of nano-vaccines, which is influenced by the route of administration, depends on the specific properties of the antigen-carrying nanoparticles. For example, the slow degradation of hard nanoparticles promotes antigen uptake when administered intravenously (I.V.), while soft nanoparticles are more efficient at stimulating antigen uptake when delivered via subcutaneous (S.C.) injection (76). However, testing of new vaccines typically begins in small animal models, which have different anatomies from humans or other animals, and as a result, the route of administration cannot be fully evaluated in these models. Thus, studies using different vaccine administration routes in small animals are insufficient for determining the most effective immunization routes for other animals or humans (77). Researchers in the studies reviewed have used various routes of administration for their candidate NP vaccines, including intramuscular (I.M.), subcutaneous (S.C.), intradermal (I.D.), intraperitoneal (I.P.), and intranasal (I.N.) (**Table 2**).

### 3.6 Immunogenicity assessment methods

To obtain “proof of concept” data supporting clinical trial development, assessing the immunogenicity of potential vaccines in animal models is essential (78). The induction of adaptive immunity can be evaluated through humoral or cellular immune responses. The most widely used methods for evaluating vaccine immunogenicity include seroconversion rates, neutralizing antibody titers, and immune cell proliferation assays (79). Additionally, the efficacy of new vaccines is typically evaluated based on protection against a lethal dose of the pathogen. As indicated in **Table 2**, the majority of the reviewed studies assessed immunogenicity using virus-neutralizing assays, immunoglobulin G (IgG) titers, and protection against lethal dose challenges. Virus-neutralizing and seroconversion assays are crucial for vaccine evaluation, as they quantify neutralizing antibodies that inhibit viral infections and indicate potential protective immunity (2). However, these assays primarily focus on antibody responses, potentially overlooking other key components of immunity, such as T-cell responses and non-neutralizing antibodies, which also contribute to protection (80, 81). Non-neutralizing antibodies enhance immunity by facilitating opsonization, activating the complement system, and promoting antibody-dependent cellular cytotoxicity (ADCC), which targets infected cells for destruction. Additionally, non-neutralizing antibodies influence immune cell activation and differentiation, shaping the overall immune response. While not directly neutralizing pathogens, these antibodies play critical roles in pathogen clearance and immune regulation (82).

Most of the recent studies also included lymphocyte proliferation assays (62, 63, 65, 68, 70, 71) and cytokine assays to further evaluate vaccine immunogenicity (52, 61, 63, 70, 83). Lymphocyte proliferation assays are essential for assessing immune responses to new vaccine candidates, as they measure the activation and expansion of T and B cells upon antigen exposure. Using techniques like radiolabeled thymidine incorporation or flow cytometry, these assays evaluate T-cell activation and differentiation into effector and memory cells (84). A robust proliferation response indicates effective immune activation and suggests strong immunogenicity (85, 86). However, these assays do not directly assess the functionality or effectiveness of the immune response, such as antibody or cytokine production. Therefore, while lymphocyte proliferation assays provide valuable information, they should be complemented with other immunological assessments for a comprehensive evaluation of vaccine efficacy (87). Cytokine assays are also crucial for evaluating immunogenicity, as they measure key cytokines that reflect immune cell activation. Cytokines like IL-12, TNF-α, IFN-γ, and IL-4 help assess the strength and type of immune response, including Th1 (cellular immunity) and Th2 (humoral immunity) responses, which are critical for vaccine efficacy (88, 89). Cytokine assays also provide insights into immune memory by detecting markers such as IL-2 and IFN-γ, which are associated with memory T-cell differentiation (90).

### 3.7 Immune response and protection

This systematic review reveals that all of the studies reviewed employed a virus-neutralizing assay, and in nearly all cases, the candidate vaccines elicited higher neutralizing antibody titers. Additionally, elevated levels of IgG, IgG1, and IgG2a were observed in immunized mice (50, 54, 60, 63, 68) (**Table 2**). The VLP-based candidate vaccine, engineered from JEV prM and E proteins in BHK-21 cells, induced higher neutralizing antibody titers compared to live attenuated vaccines (45). VLPs mimic the viral structure, enhancing antigen presentation and boosting immune responses (91). They also stimulate humoral immunity through a T-helper cell-independent pathway (92) and activate CD8+ cytotoxic T cells via the MHC class I pathway, bypassing the need for extracellular antigens (93). This process involves antigen uptake by CD8− dendritic cells and transfer to secondary lymphoid organs, where it is presented via TAP-dependent and independent pathways (94). Similarly, γ-PGA-based nanoparticles containing inactivated JEV (55) induced higher neutralizing antibody titers than live vaccines. γ-PGA stimulates innate immunity, promotes Th1 responses, and enhances cytotoxic T lymphocyte (CTL) activity (95). Its adjuvant properties further amplify the immune response by activating antigen-presenting cells and T cells, leading to increased antibody production and stronger immune memory (96).

Cytokine concentrations induced by the NP vaccines were also analyzed, with results showing elevated levels of IL-4, IL-12, IFN-α, and IFN-γ (61, 63, 70, 83). The candidate NP vaccines effectively activated both innate immune cells (dendritic cells and macrophages) and adaptive immune responses, providing robust protection against pathogens. Activation of APCs stimulates pro-inflammatory cytokine production, primarily through PRRs such as TLRs (97, 98). This activation drives the differentiation of CD4+ T cells into Th1 and Th2 subsets: IL-12 from APCs promotes Th1 differentiation and IFN-γ production, while IL-4 drives Th2 differentiation, enhancing antibody responses (32, 99, 100). NPs also stimulate type I interferon production, including IFN-α, through signaling pathways activated by viral components or adjuvants. Cytokines such as IL-12 and IL-4 create feedback loops that amplify cytokine production, with IFN-γ further stimulating IL-12 production. Enhanced antigen presentation by NPs improves T-cell activation and cytokine production, particularly for CD8+ cytotoxic and CD4+ helper T cells (101). This cross-talk between immune cells leads to a coordinated cytokine response, contributing to improved vaccine efficacy and protection (102, 103).

Furthermore, the candidate JE NP vaccines induced significant T-cell proliferation in the spleen (62, 65, 71). This immune cell proliferation is linked to the process in which, upon antigen administration, APCs present processed antigen peptides on MHC molecules, which is critical for T-cell activation. Naive T cells in lymphoid tissues encounter these antigen-MHC complexes, triggering the first signal necessary for activation. For full activation, T cells also require co-stimulatory signals from APCs, such as the interaction between CD80/CD86 on APCs and CD28 on T cells (104). Additionally, activated APCs secrete inflammatory cytokines, including IL-12, IL-6, and TNF-α, which further enhance T-cell activation and proliferation. Other cytokines, such as IFN-γ and IL-4, promote T-cell differentiation into effector subsets, increasing their proliferative capacity and the number of antigen-specific T cells in lymphoid tissues. Some of these proliferating T cells differentiate into memory T cells, which persist and provide long-term immunity, ensuring a rapid response upon re-exposure to the antigen (37). These findings suggest that NP-based candidate vaccines can effectively enhance both humoral and cellular adaptive immune responses against JEV. The next-generation NP vaccines provided 70–100% protection for immunized mice against a lethal dose of virulent JEV, despite variations in adjuvants, doses, NP types, antigens, and animal models used across studies (**Table 2**).

## 4 Discussion

Developing vaccines against viral infections is generally more straightforward than creating antiviral drugs, and vaccines are far more effective at preventing disease progression before significant damage occurs. JE is viral encephalitis for which no proven antiviral treatment exists; therefore, vaccination remains the primary strategy for effectively controlling and preventing the disease. As noted in the introduction, both live-attenuated and inactivated vaccines are utilized in several countries and play a crucial role in JE prevention (20), despite concerns regarding cost, safety, and challenges related to cross-protection (30). The high genetic and antigenic variability of the JEV complicates the development of a universally protective vaccine. JEV’s diverse genotypes, which exhibit variations in the E protein, result in differences in immune recognition, making vaccines designed for specific strains less effective against others (15, 105). For instance, while inactivated and live-attenuated vaccines show strong efficacy against genotype III, they offer reduced protection against genotype I (106, 107). This variability, along with the risk of immune escape due to antigenic mutations, presents significant challenges in developing a universal JEV vaccine (108).

To address these issues, recombinant vaccines present a promising solution, as they enable precise targeting of conserved epitopes across multiple JEV strains. Using recombinant DNA technology, specific viral proteins, such as the E protein from different strains, can be produced and purified. This reduces the risk of immune escape and facilitates the creation of multivalent vaccines that provide broader protection (31, 41). The adjuvant requirements and early degradation problems often associated with recombinant vaccines can be mitigated through nanoparticle-based delivery systems (39, 109). By encapsulating multiple epitopes from different JEV strains, nanoparticles can contribute to the development of multivalent vaccines that offer more durable and comprehensive immunity, effectively addressing cross-strain protection. Thus, the combination of recombinant vaccine technology and nanoparticle delivery systems holds considerable promise for overcoming JEV’s genetic diversity and enhancing global vaccine coverage (31). This review consolidates findings on the development of nanoparticle-based vaccines for JE, aiming to improve understanding and guide the future development of safe, effective, multivalent, and affordable vaccines. A variety of nanoparticles, including self-assembled proteins, biological polymers, synthetic compounds, and inorganic nanoparticles, are being explored as antigen carriers for JE vaccines (110, 111).

### 4.1 Self-assembled protein-based JE NP vaccines

**Figure 5:**
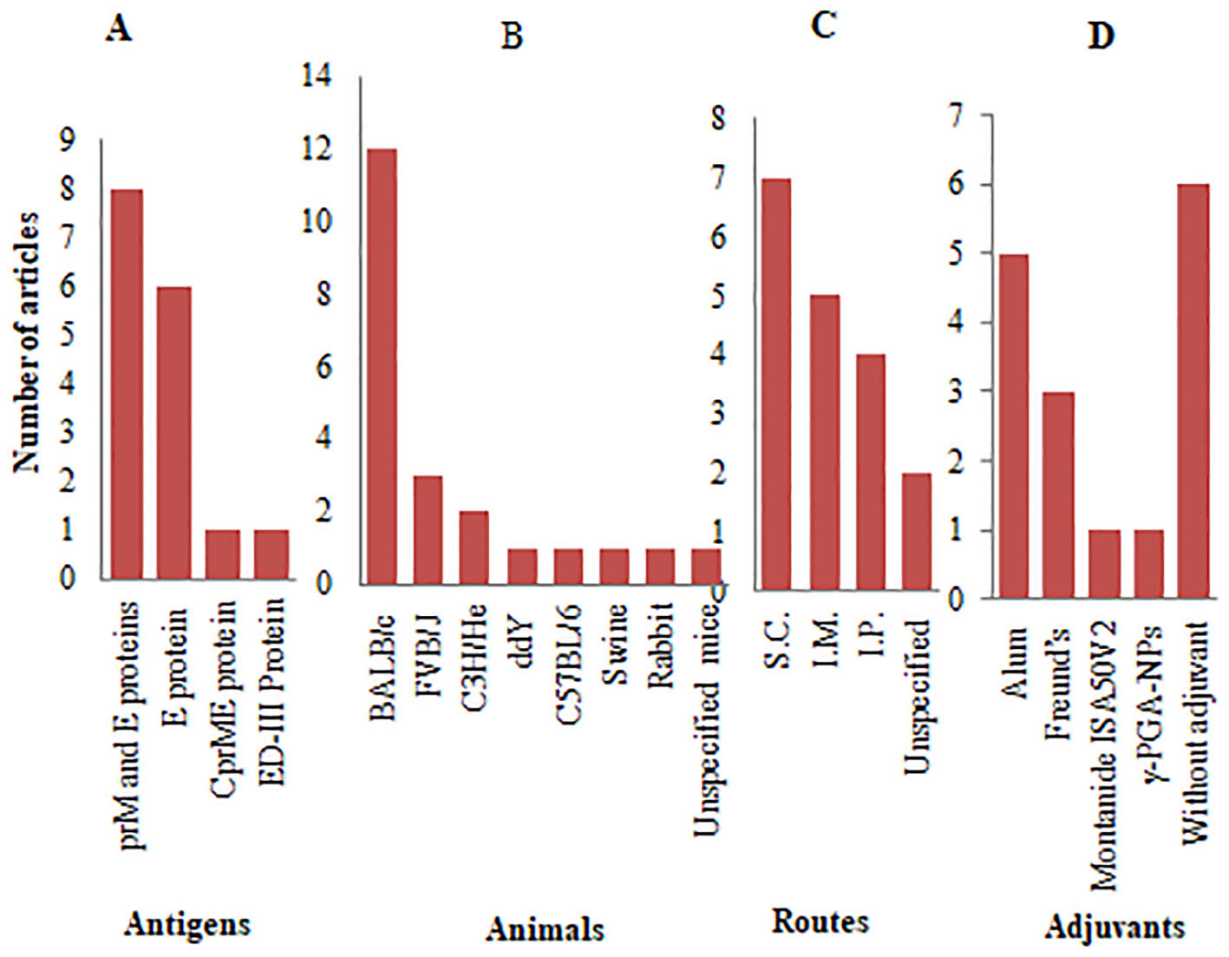
Antigenic proteins, animal models, and administration systems employed in JE VLP vaccine studies: **(A)** Types of antigenic proteins with frequency of studies; **(B)** Animal models with frequency of studies; **(C)** Routes of administration with frequency of studies; **(D)** Types of adjuvants with frequency of studies.

Over 50% of the reviewed articles focused on VLP-based vaccine development, utilizing structural JEV proteins as antigens. **Figure 5** provides an overview of the antigenic components, adjuvants, animal models, and administration routes used in JE VLP vaccine studies. Recombinant technology enables the *in vitro* production of viral structural proteins (112), leading to the formation of smaller entities known as sub-viral particles, which elicit robust innate, humoral, and cellular immune responses in both animals and humans (113). The majority of these *in vitro*-generated sub-viral particles retain the characteristics of VLPs, composed of one or more full-length viral structural proteins. In contrast, some consist of smaller sub-viral particles formed from truncated capsid proteins (114).

In conjunction with mRNA and viral vector-based vaccines, VLP technology provides an alternative platform for developing effective vaccines against major infectious diseases. Both SVPs and VLPs elicit robust immune responses by activating innate and adaptive immunity. Upon uptake by DCs and other APCs, VLPs and SVPs trigger TLR-mediated signaling pathways, promoting the release of pro-inflammatory cytokines and enhancing antigen presentation (115). Their repetitive structure efficiently activates B cells, leading to the production of high-affinity antibodies that neutralize pathogens (116). Additionally, these particles stimulate CD4+ T-helper cells, which facilitate B cell differentiation and activate CD8+ cytotoxic T cells via cross-presentation, offering strong protection against infections (117). Moreover, the particulate nature of VLPs and SVPs enhances long-term immunity by promoting the formation of memory B cells and T cells (118).

Virus-like particles (VLPs) are considerably more immunogenic due to their repetitive antigenic epitopes, which provide a more authentic signal for immune system recognition. As summarized in the main findings section (**Table 2**), VLP-based candidate vaccines, both with and without adjuvants, were able to elicit immune responses comparable to those induced by live and inactivated vaccines. Five studies—conducted by Yang et al. (68), Chang et al. (61), de Wispelaere et al. (67), Mutoh et al. (119), and Saini and Vrati (48)—demonstrated that VLPs, even without adjuvants, could induce neutralizing antibodies against JEV. In contrast, subunit vaccines typically require adjuvants and booster doses to elicit an adequate immune response. The reviewed articles also indicated that mice immunized with sub-viral particles, with or without adjuvants, developed neutralizing antibodies against JEV (53, 65).

### 4.2 Biopolymer based JE NP vaccines

Chitosan is a positively charged, biocompatible polymer that acts as a natural mucoadhesive agent. As a result, over the past decade, chitosan-derived nanoparticles (CS NPs) have gained widespread use for delivering vaccine antigens via the mucosal route (120). CS NPs enhance immune activation by promoting the uptake of antigens by DCs and macrophages, which, in turn, stimulates TLR-mediated signaling and the production of pro-inflammatory cytokines (121). In studies focused on NP vaccine development against JEV, chitosan facilitated the administration of live attenuated vaccines via the I.N. route and the DNA of prM and E protein genes via the I.D. route. The findings revealed that I.N. administration resulted in significantly higher levels of specific anti-JEV IgA. However, cytokine levels and neutralizing antibodies were markedly lower compared to those achieved via S.C. administration (83). In the case of I.D. delivery of DNA, specific antibodies were generated, conferring 100% and 50% protection with and without adjuvant, respectively (69).

Lumazine synthase (LS) is a family of enzymes that plays a crucial role in versatile vaccine delivery systems due to its oligomeric structure, which exhibits remarkable conformational stability. Lumazine synthase nanoparticles, with their self-assembling properties, facilitate the presentation of antigens to B cells and CD4+ T-helper cells, inducing antibody production and T cell responses (122). The antigens are displayed in a well-organized array on the surface of LS, creating a high local concentration of antigens. These repetitive patterns facilitate the cross-linking of B-cell receptors through an avidity effect, leading to robust immune responses (123). A study by Yao and colleagues (63) demonstrated that LS-assembled EDIII JEV protein significantly increased neutralizing antibody titers (IgG1 and IgG2a) and cytokine levels (IFN-α, IL-12, and IFN-γ). The antibodies elicited by EDIII-LS were comparable to those produced by the live attenuated vaccine (SA14-14-2) and substantially higher than those from the EDIII subunit vaccine. Both EDIII-LS and SA14-14-2 vaccinations achieved 100% protection in challenged mice, whereas only 55% of mice vaccinated with the EDIII subunit vaccine were protected.

AB5 toxins are critical virulence factors for major bacterial pathogens, consisting of a catalytic A-subunit that disrupts host functions and a B-subunit that binds to specific glycan receptors on target cell surfaces. The non-toxic B5 component of the holo-toxin (AB5) provides a pentameric scaffold for assembling antigenic proteins, mimicking the native five-fold axis (124). AB5 toxin-based nanoparticles exploit the unique ability of the AB5 toxin to bind to ganglioside receptors on host cells, leading to enhanced antigen uptake by APCs, triggering both humoral and cellular immune responses (125). Ahn et al. hypothesized that genetically fusing the B-subunit of the AB5 toxin with a viral antigenic protein would facilitate pentameric self-assembly while preserving the conformational epitopes necessary for an effective immune response. Their study led to the development of a pentameric nanoscale JEV EDIII protein vaccine using cholera toxin B (CTB) and heat-labile enterotoxin B (LTB). The results indicated that both CTB-EDIII and LTB-EDIII recombinant proteins induced high total IgG levels and similar IgG1 levels compared to the inactivated vaccine, as well as comparable neutralizing antibody titers (60).

Bio-nanocapsules, derived from bacterial Nano cellulose, also promote antigen delivery to dendritic cells (DCs), activating CD8+ cytotoxic T cells and enhancing the cross-presentation of antigens, which is crucial for effective cellular immunity (126). A bio-nanocapsule NP-based anti-JE candidate vaccine was constructed by loading the JEV E protein domain 3 onto the surface of two tandem repeats of the Z domain (ZZ-BNC) derived from *Staphylococcus aureus* protein A. However, the protection conferred against lethal JEV challenges in immunized mice was relatively low (44.4% without adjuvant and 70% with alum adjuvant), while the inactivated JE vaccine provided complete protection (127).

Poly (γ-glutamic acid) (γ-PGA) is a biopolymer composed of repeating units of D- and L-glutamic acid, naturally polymerized via γ-amide bonds. γ-PGA possesses excellent biocompatibility and stability, which enhance the adjuvant properties of vaccines by improving immune cell recruitment and antigen persistence, leading to strong long-term immunity (128). It is non-toxic and biodegradable, allowing for easy uptake by DCs, which leads to cytokine secretion that enhances Th1 immune responses and boosts cytotoxic T lymphocyte (CTL) activity (96). Okamoto et al. evaluated the adjuvanticity of γ-PGA NPs in combination with an inactivated JEV vaccine, demonstrating that a single dose of the JE vaccine with γ-PGA NPs significantly enhanced neutralizing antibody titers. This resulted in all immunized mice surviving a normally lethal JEV infection, whereas only 50% of those receiving a single dose of the JE vaccine without γ-PGA NPs survived (55).

### 4.3 Synthetic and inorganic NP-based JE vaccines

Lipid nanoparticles (LNPs) represent a groundbreaking class of nanoparticles with immense potential for the delivery of nucleic acids and eliciting robust immune responses. LNPs are efficiently taken up by antigen-presenting cells (APCs) via endocytosis or phagocytosis, leading to the release of the encapsulated antigen in the endosome, where it is processed and presented on major histocompatibility complex (MHC) class I and II molecules. This stimulates both CD8+ cytotoxic T cells and CD4+ helper T cells (129). The antigen presentation activates the adaptive immune response, leading to the production of neutralizing antibodies by B cells and the elimination of infected cells by CD8+ T cells. Additionally, LNPs interact with pattern recognition receptors (PRRs), such as TLR7 and TLR8, on APCs, which recognize viral RNA. This interaction triggers the production of inflammatory cytokines, including IL-6, TNF-α, and type I interferons, enhancing APC maturation and promoting a stronger immune response (130). Furthermore, the particulate nature of LNPs serves as an adjuvant, boosting the immune response by mimicking viral membranes, which improves both the delivery of antigens and the activation of immune cells (131). LNPs also facilitate the formation of memory B cells and T cells, ensuring long-term immunity and protection upon re-exposure to the pathogen (130). This combination of efficient antigen delivery, activation of both innate and adaptive immunity, and adjuvant effects makes LNPs highly effective in vaccines, particularly for viral infections (132).

The promise of LNPs has been underscored by the recent emergency use authorization (EUA) granted by the US FDA for two mRNA-based SARS-CoV-2 vaccines: mRNA-1273 (Moderna) and BNT162b2 (Pfizer-BioNTech). Consequently, researchers involved in vaccine development have become increasingly interested in the mRNA-LNP vaccine platform (129). Wide arrays of mRNA-encapsulated LNPs are currently under investigation in clinical settings for various applications, including hereditary disorders, viral infections, and cancer (28, 133). In a similar vein, Chen et al. developed an mRNA vaccine encoding the JEV prM and E proteins, rigorously evaluating its immunogenicity and protective efficacy. Their findings demonstrated that mRNA immunization could elicit robust JEV-neutralizing antibodies and potent CD8+ T-cell responses, effectively safeguarding mice against JEV infection (62).

Polyethylene glycol (PEG) is a versatile polyether molecule that is non-ionic and has multiple applications within the pharmaceutical industry. PEG is commonly used to modify the surface characteristics of nanoparticles (NPs) and enhance their molecular weight (MW) (134). PEG improves the bioavailability of antigens by reducing clearance through the reticuloendothelial system (RES), allowing for more efficient delivery to APCs. Once internalized, PEG NPs promote the activation of PRRs, including TLRs, on the surface of APCs. This triggers intracellular signaling pathways, such as NF-κB and MAPK, leading to the release of pro-inflammatory cytokines like IL-1β, IL-6, and TNF-α, which help recruit additional immune cells to the site of antigen presentation (135, 136). After processing the antigen, APCs present it on MHC class I and II molecules, activating CD8+ cytotoxic T cells and CD4+ helper T cells, respectively. PEGylation also serves as an adjuvant, improving the overall immune response by enhancing antigen delivery, stabilizing the vaccine components, and providing controlled release (137). Moreover, PEG coating can mitigate immune responses against the nanoparticles themselves, which could otherwise reduce the efficacy of repeated vaccinations (136, 138). Hunt et al. utilized PEG to precipitate purified recombinant JEV protein particles. Immunogenicity evaluations revealed that PEG-precipitated recombinant proteins, in conjunction with Freund’s incomplete adjuvant, induced higher neutralizing antibody titers against JEV (57).

Well-functionalized gold nanoparticles (AuNPs) are among the most promising nanomaterials for the next generation of vaccines (134, 139). Their reliable surface functionalization, biocompatibility, customizable size and shape, and unique optical properties have generated considerable interest in the field of vaccinology. Upon administration, AuNPs are taken up by immune cells, including DCs, macrophages, and B cells, via receptor-mediated endocytosis. The nanoparticles are then transported to lymph nodes, where they present antigens to T cells, initiating the adaptive immune response (88, 140). AuNPs also interact with immune cells through pattern recognition receptors (PRRs), such as Toll-like receptors (TLRs) and C-type lectin receptors (CLRs), which activate the innate immune system and trigger the release of pro-inflammatory cytokines (TNF-α, IL-1β, IL-6). These cytokines recruit additional immune cells and create a pro-inflammatory environment that supports adaptive immune activation (88). Furthermore, gold nanoparticles act as adjuvants, enhancing the immune response to co-administered antigens by increasing both the strength and duration of the response (88, 141, 143). AuNPs also stimulate the production of type I interferons (IFN-α/β) and promote the activation of B cells, CD4+ helper T cells, and CD8+ cytotoxic T cells, which are critical for both humoral and cell-mediated immunity (139, 142).

Nucleic acid strands can be covalently bonded to the cores of gold nanoparticles (typically 13–15 nm) through thiol moieties (144–146). This innovative strategy applies to both DNA and siRNA, which can be directly conjugated to gold cores or to polymer-modified gold cores (147). Two decades ago, Zhao and colleagues conducted a JE immunization experiment using colloidal gold to inoculate plasmid DNA encoding the prM and E proteins. It remains unclear whether the gold served primarily as a carrier or as an adjuvant in this case. Nevertheless, this immunization approach facilitated a more rapid production of specific anti-JEV neutralizing antibodies via intravenous (I.V.) and intramuscular (I.M.) routes compared to alternative methods. Both active and passive (anti-JEV sera) immunization conferred 100% protection against JEV challenges at 10^5^ LD50 (47).

## 5 Conclusion

Epidemiological data indicate that JEV is steadily expanding into new territories across Europe and Africa, in addition to its established presence in the Asia-Pacific region, with the potential for global dissemination in the near future (7). While the widespread use of traditional vaccines has successfully reduced the incidence of JE among children in endemic areas, there has been a noticeable rise in cases among adults (148, 149). JEV also poses significant threats to animal health and the economy, as it not only causes illness and mortality in livestock but also leads to substantial financial losses for farmers due to reduced productivity. As a result, there is an ongoing demand for effective and safe vaccines that can be produced at scale with minimal financial investment. In response, scientists are actively exploring innovative approaches to develop novel JE vaccines. This review highlights the growing array of nanoparticle-based vaccine candidates that have emerged from the dedicated efforts of researchers. In the foreseeable future, these promising candidates could pave the way for next-generation JE vaccines for both humans and animals as they progress through subsequent stages of the vaccine development pipeline.

### 5.1 Limitations

The quality of the studies included in this review varied considerably, with some exhibiting methodological weaknesses that could impact the robustness of the findings. The heterogeneity among the studies—particularly regarding administration systems (route, dose, booster immunization, and use of adjuvants) and the methods for evaluating immunogenicity—may limit the comparability of results and their interpretation. Additionally, the review was confined to studies published in English, which may have excluded valuable research published in other languages that could offer further insights.

### 5.2 Future directions for JEV vaccine development

Future research on JEV vaccines should address key challenges to improve efficacy and broader applicability. A major focus should be developing broad-spectrum vaccines that provide cross-protection against all five JEV genotypes. Enhancing antigen presentation systems and optimizing antigen combinations could also boost vaccine effectiveness. Additionally, exploring cross-protection against other flaviviruses and improving immunization strategies for broader flavivirus threats will be critical. Tackling these issues will be essential for advancing JEV vaccine development and preparing for emerging flavivirus-related diseases.

## Data Availability

The raw data supporting the conclusions of this article will be made available by the authors, without undue reservation.
